# The influence of cell phenotype on collective cell invasion into the extracellular matrix

**DOI:** 10.1007/s11538-025-01560-9

**Published:** 2025-12-26

**Authors:** Yuan Yin, Sarah L. Waters, Ruth E. Baker

**Affiliations:** Mathematical Institute, University of Oxford, Oxford, UK

## Abstract

Understanding the interactions between cells and the extracellular matrix (ECM) during collective cell invasion is crucial for advancements in tissue engineering, cancer therapies, and regenerative medicine. This study focuses on the roles of contact guidance and ECM remodelling in directing cell behaviour, with a particular emphasis on exploring how differences in cell phenotype impact collective cell invasion. We present a computationally tractable two-dimensional hybrid model of collective cell migration within the ECM, where cells are modelled as individual entities and collagen fibres as a continuous tensorial field. Our model incorporates random motility, contact guidance, cell–cell adhesion, volume filling, and the dynamic remodelling of collagen fibres through cellular secretion and degradation. Through a comprehensive parameter sweep, we provide valuable insights into how differences in the cell phenotype, in terms of the ability of the cell to migrate, secrete, degrade, and respond to contact guidance cues from the ECM, impacts the characteristics of collective cell invasion.

**Supplementary Information:**

The online version contains supplementary material available at 10.1007/s11538-025-01560-9.

## Introduction

Collective cell invasion is a fundamental, tightly coordinated process involving both migration and proliferation, and it takes place throughout development, wound healing, and cancer metastasis (Friedl and Gilmour 2009; Ilina and Friedl 2009; Mayor and Etienne-Manneville 2016; Stock and Pauli 2021). This coordinated invasion is driven by cell–cell interactions and extrinsic cues provided by the surrounding microenvironment, with the extracellular matrix (ECM) playing a pivotal role (Yue 2014). The ECM is a complex and dynamic macromolecular network composed of various proteins, such as collagen, fibronectin, and glycosaminoglycans (Karamanos et al. 2021). Collagen fibres are the predominant component of the ECM, and are essential for maintaining the mechanical integrity of tissues under stress (Rezvani Ghomi et al. 2021). Cells actively remodel the ECM by secreting, degrading, and reorganising its components as they migrate (Frantz et al. 2010). This dynamic interplay between cells and the ECM underlies many physiological and pathological processes, however its huge complexity means that it remains challenging to fully characterise using experiments alone. An additional complicating factor is that cells exhibit phenotypic heterogeneity, reflected in their response to their local biomechanical environment, metabolic activity, protrusive activity and connectivity patterns (Cheung et al. 2013; Beunk et al. 2022; Agnihotri and Zadeh 2015; Haeger et al. 2015; Friedl and Mayor 2017; Wolf et al. 2007).

Mathematical modelling offers a powerful approach to systematically investigate the roles of various cell–cell and cell–ECM interactions in driving collective cell migration, and to explore how differences in cell phenotype drive patterns of collective cell invasion. The overarching aim of this work is to develop a computationally tractable model of collective cell migration that includes both cell–cell interactions, such as adhesion and volume filling, and cell–ECM interactions, which lead to contact guidance and ECM evolution, and use it to explore how they together drive collective cell migration. In particular we focus on elucidating how differences in the cell phenotype, in terms of their ability to migrate, secrete, degrade, and interact with the ECM, impact collective cell invasion signatures.

### Cell–ECM interactions during collective migration

The ECM is not merely a passive mechanical support; it provides a dynamic environment that facilitates biochemical signalling, influencing cell activities such as migration, differentiation, and proliferation (Yue 2014). Comprehensive reviews of ECM components, properties, and roles in cellular functions can be found in Kyriakopoulou et al. (2023), Padhi and Nain (2020), and Walma and Yamada (2020). Among the mechanisms by which the ECM influences cell motility is haptotaxis, where cells migrate along gradients of ECM-bound molecules. Haptotaxis a key process in tissue regeneration and cancer invasion (Pekmen and Yirmili 2024; Sfakianakis et al. 2020). Another mechanism is durotaxis, where cells migrate towards regions of stiffer ECM, a process linked to cancer metastasis (Espina et al. 2022). Finally, contact guidance is another important cell–ECM mechanism—here, collagen fibre alignment provides directional cues that reorients cell migration paths (Pamonag et al. 2022; Kim et al. 2021). Experimental evidence demonstrates a key role for contact guidance in wound healing, where fibroblasts migrate by aligning with collagen fibres at the wound site (Lackie 2012). There is also experimental evidence that contact guidance modulates the speed of cell migration (Doyle et al. 2009).

The interaction between cells and the ECM is not unidirectional; cells actively remodel the ECM by secreting, degrading, and reorganising its components (Frantz et al. 2010). Fibroblasts, for example, can secrete collagen fibres that are aligned in the direction of their movement, thereby reinforcing pathways for future migration (McDougall et al. 2006). Cells also employ matrix metalloproteinases (MMPs) to degrade ECM components, a process that is particularly well-studied in the context of cancer invasion and metastasis (Reunanen and Kähäri 2013). Additionally, cells interact mechanically with the ECM by exerting forces that remodel its structure. For instance, fibroblasts exert traction forces on newly formed granulation tissue, which increases its rigidity. This increasing stiffness strengthens cell–ECM contacts, encourages the formation of intracellular contractile stress fibres, drives fibroblast differentiation into proto-myofibroblasts, and facilitates migration (Hinz and Gabbiani 2003). Recent work also highlights that fibroblasts exhibit mechanical memory when subjected to prolonged stretching: moderate stretching activates fibroblasts, while excessive stretching inhibits them (Weihs 2025).

The intricate bidirectional feedback between cells and the ECM during collective cell invasion presents a significant challenge in disentangling how specific mechanisms drive certain dynamics and how these dynamics, in turn, influence each other. In this study, we aim to gain insights through the development of a tractable mathematical model to address the overarching question of how the bidirectional interactions between cells and the ECM drive the dynamics of collective cell migration while simultaneously shaping the architecture of the ECM, and how these patterns of collective invasion are altered in cell populations with different phenotypes.

### Mathematical models of ECM–cell interactions

A key challenge in mathematical modelling of collective cell invasion is striking a balance between biological realism and computational tractability. Models must incorporate sufficient mechanistic detail to yield meaningful biological conclusions while remaining simple enough to allow for systematic parameter exploration. A broad range of modelling approaches have been developed to study cell–ECM interactions, as reviewed in Crossley et al. (2024).

Of particular relevance to this study are several modelling frameworks that focus on the interplay between migrating cells and components of the ECM. Dallon et al. (1999) modelled cells as discrete agents and collagen fibres as a continuous bidirectional vector field. The direction of cell migration is determined by two factors: the tendency of a cell to persist in its prior path, and the directional cues from the surrounding collagen fibres. To account for the presence of multiple collagen fibres at each point in space, Hillen (2006) and Painter (2009) introduced more complex models in which collagen fibres are represented by a probability distribution function that varies with orientation, space, and time. Cell movement is described by a velocity-jump process, formulated using a transport equation in which contact guidance depends on the surrounding collagen distributions. Though they capture many of the salient features of cell invasion into the ECM, both models neglect direct cell–cell interactions.

Cumming et al. (2010) extended the approach of Dallon et al. (1999) to include additional complexity of the ECM, developing a six-species hybrid model that considers the dynamics of fibrin, collagen, macrophages, fibroblasts, transforming growth factor-$\beta$ (TGF-$\beta$) and tissue plasminogen activator. Collagen fibres dynamics are represented by a continuous tensorial field that captures fibre orientation and density, with computational tractability retained by decomposing the tensorial field into the sum of its (orthogonal) eigenvectors’ outer products. Each cell is represented as a circular disc, and its migration is influenced by mechanisms such as contact guidance and chemotaxis. Although it captures many features of the ECM, as well as cell signalling, the simple representation of cells in this model precludes study of how cell–cell interactions, such as cell–cell adhesion and volume filling, impact collective migration.

Recent efforts have increased model complexity to study specific biological phenomena. For instance, to explore complex ECM patterning in both normal and pathological tissues, Wershof et al. (2019) modelled cells as diamond-shaped agents and the ECM as an orientation field on a grid with eight possible directions. Their model incorporates random motion, cell–cell and ECM guidance, collagen secretion, degradation, and rearrangement. The authors demonstrate how basic rules governing fibroblast–ECM interactions can give rise to complex tissue patterns. Poonja et al. (2023) developed an off-lattice hybrid model, where cells are represented as discrete agents and the ECM as a unit vector field, to study contact guidance and cell-generated forces on the ECM, which lead to different ECM fibril alignments and tumour-associated collagen signatures. To predict neovessel guidance during angiogenesis, LaBelle et al. (2023) developed computational models where collagen is represented as a deformable three-dimensional ellipsoidal fibril distribution, accounting for the interplay between collagen orientation, anisotropy, density, and vessel alignment. While these models provide a robust framework for incorporating diverse biophysical phenomena, their complexity often limits the systematic parameter studies that are necessary to isolate and understand specific mechanisms.

### Aims and outline

The primary aim of this work is to develop a computationally tractable mathematical model that focuses exclusively on the role of ECM-generated contact guidance, enabling detailed exploration of how collagen fibres influence cell migration and *vice versa*, whilst at the same time incorporating biologically realistic cell–cell interactions, such as volume filling and cell–cell adhesion. We perform a comprehensive model parameter sweep to explore how collective cell invasion signatures are shaped by cell phenotype.

The minimal model we develop provides advances over existing models by integrating well-established, individual-based mathematical representations of cell–cell interactions, such as cell–cell adhesion and volume filling, with the continuous tensorial description of collagen fibres proposed by Cumming et al. (2010). We focus on the role of contact guidance in influencing cell motility, and the impact of cells in remodelling the collagen field. For simplicity, we assume that contact guidance modulates the direction of cell migration without affecting the speed, though this feature could easily be included in future iterations of the model. A systematic exploration of the model predictions across different regions of parameter space enables us to explore how cell phenotype impacts invasion dynamics.

The structure of the paper is as follows: In Section 2, we outline the mathematical model and in Section 3 we detail the computational implementation. Section 4 presents our findings on how different mechanisms, separately and collectively, give rise to various cell and collagen fibre dynamics. Finally, we discuss the results and future directions in Section 5.

## Mathematical model

**Fig. 1:**
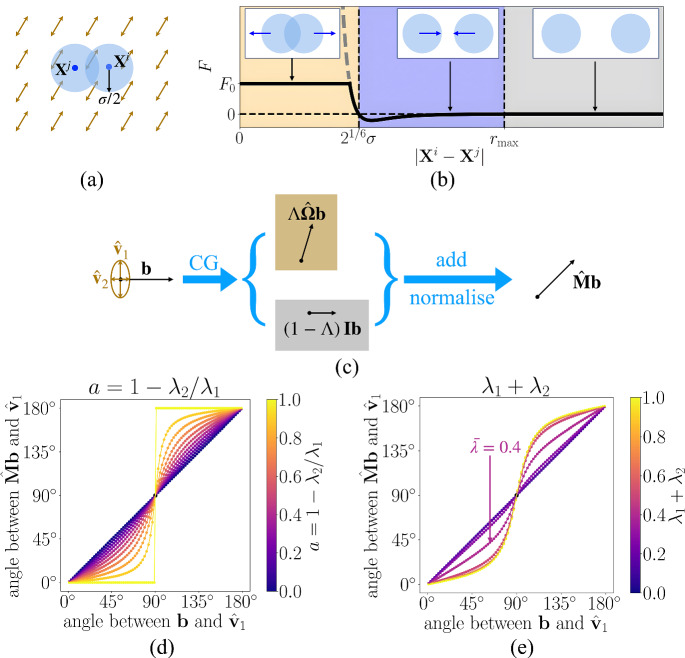
**Schematic illustration of the model.** (a) Cells *i* and *j* are depicted as blue circular discs with radius $\sigma /2$ centred at $\textbf{X}^i$ and $\textbf{X}^j$, respectively. They sit on top of collagen fibres, where major fibre orientation $\hat{\textbf{v}}_1$ and the associated area fraction $\lambda _1$ are visualised using brown arrows. (b) The magnitude of the pairwise interaction force is given by the function $F(|\textbf{X}^i- \textbf{X}^j|)$ defined in Equation (7). Orange region: short-range repulsion due to the effects of volume filling; blue region: mid-range adhesion; grey region: no cell–cell interactions. The dashed grey line represents the force arising from the Lennard-Jones potential, and $F_0>0$ sets the maximum repulsive force used in the model. (c) Schematic diagram of how the contact guidance matrix $\hat{\textbf{M}}$ reorients vector $\textbf{b}$ given vertical $\hat{\textbf{v}}_1$ and horizontal $\hat{\textbf{v}}_2$. (d),(e) Illustration of the mechanisms of contact guidance indicating how $\hat{\textbf{M}}$, defined in Equation (8), reorients a vector $\textbf{b}$, given different anisotropy degrees, *a* (d), and total area fractions, $\lambda _1+\lambda _2$ (e). In (d), $\lambda _1+\lambda _2=1.0$ and in (e), $a=0.8$. In (e), the arrow indicates the strength of contact guidance at the critical total fibre area fraction $\bar{\lambda }=0.4$

We formulate a two-dimensional hybrid mathematical model of collective cell motility mediated by collagen fibres. Cells are modelled as discrete agents migrating on top of collagen fibres, and the collagen fibres are modelled via a continuous fibre field. The model incorporates the following key mechanisms: random cell motility and cell–cell interactions, both modulated by cell–fibre interactions; cell proliferation; and collagen fibre secretion and degradation by cells. We introduce the representation of cells and collagen fibres in Section 2.1, and the equations determining their evolution are given in Section 2.2. Throughout we adopt a two-dimensional Cartesian coordinate system denoted by $\textbf{x}:=\left( x, y\right) ^\textrm{T}$ with time represented by the independent variable *t*. Key variables and parameters are summarised in Tables 1 and 2, respectively, and a schematic illustration of the model can be found in Figure 1.

**Table 1:** Key variables in the model. Hats are used to denoted unit vectors and normalised (length-preserving) tensors (see Supplementary Information Section S1)

**Variable**	**Description**
*N*(*t*)	number of cells at time *t*
$\textbf{X}^i(t)$	position of cell *i*
$\textbf{u}_\textrm{ave}^i(\textbf{X}^i, t; m)$	average velocity of cell *i* during the time interval $[t-m,t]$
$\boldsymbol{\xi }^i(t)$	random stochastic force on cell *i*
$\textbf{F}(\textbf{X}^i-\textbf{X}^j)$	pairwise interaction force imposed on cell *i* by cell *j*
$\beta \left( \textbf{X}^i; \sigma \right)$	number of cells within a distance of $2^{1/6}\sigma$ of cell *i*
$P_\textrm{p}(\textbf{X}^i; \Delta _0, \Delta t, \sigma )$	probability of proliferation for cell *i* per time step $\Delta t$
$\mathbf {\Omega }(\textbf{x}, t)$	collagen fibre orientation tensor
$\hat{\textbf{v}}_1(\textbf{x}, t)$	major collagen fibre orientation
$\hat{\textbf{v}}_2(\textbf{x}, t)$	minor collagen fibre orientation
$\lambda _1(\textbf{x}, t)$	area fraction of collagen fibres in the direction of $\hat{\textbf{v}}_1$
$\lambda _2(\textbf{x}, t)$	area fraction of collagen fibres in the direction of $\hat{\textbf{v}}_2$
$a\left( \textbf{x}, t\right)$	collagen fibre anisotropy degree
$\hat{\textbf{M}}(\textbf{X}^i, t)$	contact guidance matrix for cell *i*
$\omega \left( \textbf{X}^i, \textbf{x}; \sigma , \omega _0\right)$	weight function describing cell *i*’s impact on collagen fibres

**Table 2:** Key parameters in the model, represented by $\mathbf {\Theta }=\left( G, T, \ldots , s, d\right) ^T$. Values not obtained from the literature are selected to illustrate the results presented in Section 4

**Parameter **$\mathbf {\Theta }$	**Description**	**Value**
*G*	domain of interest	$[0, 360] \times [0, 360]$ $\mu \text {m}^2$
		$[0, 540] \times [0, 540]$ $\mu \text {m}^2$
*T*	final time	3600 min, 5400 min
$\Delta t$	numerical time step	1 min
*D*	cell diffusion coefficient	0.3 $\mu \text {m}^2\text { min}^{-1}$
$\epsilon$	magnitude of pairwise interactions	0.1 $\mu \text {m}^2 \text { min}^{-1}$
$\sigma$	cell diameter	12 $\mu$m (Freitas 1999)
$F_0$	maximum repulsion	2.4 $\mu \text {m} \text { min}^{-1}$
$r_\textrm{max}$	range of cell–cell interactions	36 $\mu$m (Matsiaka et al. 2019)
$\Delta _0$	default cell cycle length	1440 min (Seaman et al. 2015)
$\bar{\lambda }$	half-maximal contact guidance	0.4
$\gamma$	steepness of transition around $\bar{\lambda }$	10
*m*	length of the averaging window	300 min
*s*	collagen fibre secretion rate	[0, 0.5] $\text {min}^{-1}$
*d*	collagen fibre degradation rate	[0, 0.5] $\text {min}^{-1}$

### Representations for cells and collagen fibres

Cells are modelled as point particles, with the centre of cell *i*, for $i=1,\ldots ,N(t)$, located at position $\textbf{X}^i(t)\in G\subseteq \mathbb {R}^2$, where *N*(*t*) is the number of cells at time *t* and *G* is the domain of interest. Cells interact via a pairwise interaction potential that captures both cell–cell adhesion and the fact that cells have finite volume (Section 2.2.1 and Figure 1(a),(b)).

The distribution of collagen fibres is represented by the tensor field $\mathbf {\Omega }(\textbf{x},t)$, where $\mathbf {\Omega }$ is symmetric and positive semi-definite. Following Cumming et al. (2010), $\mathbf {\Omega }(\textbf{x},t)$ takes the form

1 $$\begin{aligned} \mathbf {\Omega }(\textbf{x}, t) = \frac{1}{\pi }\int _{0 }^{\pi } \hat{\textbf{u}}(\theta )\hat{\textbf{u}}^\textrm{T}(\theta )\rho (\theta , \textbf{x}, t) \textrm{d} \theta . \end{aligned}$$

Here $\hat{\textbf{u}}(\theta )=\left( \cos \theta , \sin \theta \right) ^\textrm{T}$ denotes a unit vector at angle $\theta$ with respect to the positive *x*-axis, and $\rho (\theta ,\textbf{x},t)\in [0, 1]$ is the area fraction occupied by collagen fibres, which we use to represent collagen fibre density, with orientation $\theta$ at position $\textbf{x}$ and time *t*. Note that collagen fibres are bidirectional^1^ (Dallon et al. 1999; Hillen 2006) so that $\theta \in [0, \pi )$. To enable computational tractability, we follow Cumming et al. (2010) and represent $\mathbf {\Omega }(\textbf{x}, t)$ in a diagonalised form:

2 $$\begin{aligned} \mathbf {\Omega }(\textbf{x}, t) = \lambda _1 \hat{\textbf{v}}_1\hat{\textbf{v}}_1^\textrm{T} + \lambda _2 \hat{\textbf{v}}_2\hat{\textbf{v}}_2^\textrm{T}, \end{aligned}$$

where $\hat{\textbf{v}}_{1, 2}(\textbf{x}, t)$ are the orthonormal eigenvectors of $\mathbf {\Omega }$ which capture the major and minor collagen fibre orientations, respectively, and $\lambda _{1}(\textbf{x}, t)\ge \lambda _{2}(\textbf{x}, t)\in [0, 1]$ are the associated eigenvalues which represent the area fractions of collagen fibres in directions $\hat{\textbf{v}}_1$ and $\hat{\textbf{v}}_2$, respectively. Note that for the total area fraction, which represents collagen fibre density, we have

3 $$\begin{aligned} 0\le \lambda _1\left( \textbf{x}, t\right) + \lambda _2\left( \textbf{x}, t\right) = \frac{1}{\pi }\int _{0}^{\pi }\rho \left( \theta , \textbf{x}, t\right) \text {d}\theta \le 1. \end{aligned}$$

We define the anisotropy degree as $a:=1-\lambda _2/\lambda _1\in [0,1]$, where $a=1$ corresponds to perfect collagen fibre alignment along $\hat{\textbf{v}}_{1}$, and $a=0$ to a perfectly isotropic collagen fibre distribution.

### System dynamics

In describing cell dynamics we follow standard modelling assumptions and neglect inertial effects (Martinson et al. 2023; Marchello et al. 2024; Brückner and Broedersz 2024; Vargas et al. 2020; Cai et al. 2016). We describe cell and collagen fibre dynamics via the system of differential equations of the form

4a $$\begin{aligned} \frac{\textrm{d}\textbf{X}^i}{\textrm{d}t}&= \textbf{f}\left( \textbf{X}^1, \textbf{X}^2, \ldots , \textbf{X}^{N(t)}, \mathbf {\Omega };\mathbf {\Theta }\right) \text { and} \, \textbf{X}^i(t=0)=\textbf{X}^i_0 \, \text { for } i = 1, \ldots , N(t), \end{aligned}$$

4b $$\begin{aligned} \frac{\partial \mathbf {\Omega }}{\partial t}&= \textbf{g}\left( \textbf{X}^1, \textbf{X}^2, \ldots , \textbf{X}^{N(t)}, \mathbf {\Omega };\mathbf {\Theta }\right) \text { and } \, \mathbf {\Omega }(\textbf{x}, t=0) = \mathbf {\Omega }_0(\textbf{x}). \end{aligned}$$

The model is defined for $\textbf{x}, \textbf{X}^i \in G$, and $\mathbf {\Theta }$ is a vector of model parameters (see Table 2). The function $\textbf{f}$ captures cell dynamics due to random motion and cell–cell interactions, both influenced by contact guidance from the surrounding collagen fibres. The function $\textbf{g}$ captures the remodelling of collagen fibres via degradation and secretion by surrounding cells. In Sections 2.2.1–2.2.3 we are provide specific functional forms for $\textbf{f}$ and $\textbf{g}$.

#### Cell dynamics

The equation of motion for each cell $i=1,\ldots ,N(t)$ is given by Equation (4a) with

5 $$\begin{aligned} \textbf{f}\left( \textbf{X}^1, \textbf{X}^2, \ldots , \textbf{X}^{N(t)}, \mathbf {\Omega };\mathbf {\Theta }\right) =\hat{\textbf{M}}\left( \textbf{X}^i,t\right) \left[ \boldsymbol{\xi }^i+ \sum _{j=1,j\ne {i}}^{N(t)}\textbf{F}\left( \textbf{X}^i-\textbf{X}^j\right) \right] , \end{aligned}$$

where the $2\times 1$ vector $\boldsymbol{\xi }^i$ models random motility, the $2\times 1$ vector $\textbf{F}\left( \textbf{X}^i-\textbf{X}^j\right)$ captures the pairwise interactions between cell *i* and cell *j*, and the $2\times 2$ normalised (length-preserving) matrix $\hat{\textbf{M}}$ models the modulation of cell motility due to contact guidance from the underlying collagen fibres.

**Random motility and cell–cell interactions.** We assume the random motion of cell *i* can be captured by a stochastic force $\boldsymbol{\xi }^i$ (Matsiaka et al. 2019; Nava-Sedeño et al. 2017; Porta and Zapperi 2019; Berg 1993). We implement $\boldsymbol{\xi }^i$ by sampling from a Gaussian distribution with zero mean and variance $2D/\Delta t$, where $D>0$ is the macroscopic cell diffusion coefficient and $\Delta t>0$ is the numerical time step.

The pairwise interaction force experienced by cell *i* as a result of interactions with cell *j* is of the form

6 $$\begin{aligned} \textbf{F}\left( \textbf{X}^i-\textbf{X}^j\right) = F\left( |\textbf{X}^i-\textbf{X}^j|\right) \frac{\textbf{X}^i- \textbf{X}^j}{|\textbf{X}^i - \textbf{X}^j|}, \end{aligned}$$

where $F\left( |\textbf{X}^i-\textbf{X}^j|\right)$ is the magnitude of the force, which acts in the direction $\textbf{X}^i- \textbf{X}^j$. We use the Lennard-Jones potential (Wang et al. 2020; Lennard and Jones 1924) so that

7 $$\begin{aligned} \begin{aligned} F = {\left\{ \begin{array}{ll} \displaystyle \min \left\{ 24\epsilon \sigma ^6\left[ \frac{2\sigma ^6}{|\textbf{X}^i - \textbf{X}^j|^{13}} - \frac{1}{|\textbf{X}^i - \textbf{X}^j|^7}\right] , \; F_0\right\} , \quad & 0 < |\textbf{X}^i - \textbf{X}^j| \le r_\textrm{max},\\ \displaystyle 0, \quad & |\textbf{X}^i - \textbf{X}^j| > r_\textrm{max}. \end{array}\right. } \end{aligned} \end{aligned}$$

Here $\sigma >0$ is a measure of the cell diameter, and $\epsilon >0$ regulates the magnitude of the pairwise interaction forces. The additional term $F_0>0$ prevents *F* from approaching infinity as $|\textbf{X}^i - \textbf{X}^j|\rightarrow 0$ and, in line with previous works, we take $r_\textrm{max}=3\sigma >0$ (Matsiaka et al. 2019).

**Contact guidance.**

To model contact guidance, we take

8 $$\begin{aligned} \textbf{M}(\textbf{X}^i, t)= \Lambda \left( \lambda _1+\lambda _2\right) \hat{\mathbf {\Omega }}\left( \textbf{X}^i, t\right) + \left( 1-\Lambda \left( \lambda _1+\lambda _2\right) \right) \textbf{I}, \end{aligned}$$

where $\textbf{I}$ is the $2 \times 2$ identity matrix and $\hat{\mathbf {\Omega }}$ is the normalised version of $\mathbf {\Omega }$, as defined in Supplementary Information Equation (15). We then apply $\hat{\textbf{M}}$ (the normalised version of $\textbf{M}$, defined in the same way—see Supplementary Information Section S1) to the right-hand side of Equation (5) to ensure that contact guidance impacts only the direction of motion, and not cell speed.

The strength of contact guidance is weighted by a function $\Lambda$ which depends on the total area fraction of collagen fibres, $\lambda _1+\lambda _2$, and is taken to be

9 $$\begin{aligned} \Lambda (\lambda _1+\lambda _2):=\frac{h\left( \lambda _1+\lambda _2\right) -h(0)}{h(1) -h(0)},\text { where } h(x):=\frac{1}{2} \left( \tanh \left( \gamma \left( x-\bar{\lambda }\right) \right) +1\right) .\nonumber \\ \end{aligned}$$

The parameter $\bar{\lambda }\in [0,1]$ represents the critical value of $\lambda _1+\lambda _2$ at which the strength of contact guidance is half maximal, and $\gamma > 0$ controls the steepness of the transition around $\bar{\lambda }$.

The choice of $\Lambda$ ensures key properties are satisfied: when the space is saturated with collagen fibres ($\lambda _1+\lambda _2=1$), $\Lambda = 1$ and $\hat{\textbf{M}}=\hat{\mathbf {\Omega }}$, and the impact of contact guidance is maximal; whereas in the absence of collagen fibres, $\Lambda = 0$ and $\hat{\textbf{M}}=\textbf{I}$ so that the cells experience no contact guidance. Figure 1(c) visualises how $\hat{\textbf{M}}$ reorients any $2 \times 1$ vector $\textbf{b}$, and Figure 1(d)–(e) compares the angle between $\textbf{b}$ and $\hat{\textbf{v}}_1$ (the major orientation of collagen fibres) both with and without contact guidance. As shown in Figure 1(d), $\hat{\textbf{M}}$ automatically encodes the anisotropy information *a*. As *a* approaches zero (isotropic collagen fibres), $\hat{\textbf{M}}$ tends towards the identity matrix, $\textbf{I}$, and there is no contact guidance. Moreover, Figure 1(e) indicates that there is a switch between strong and weak contact guidance around $\lambda _1+\lambda _2=\bar{\lambda }$, as expected. Note that Figure 1(d)–(e) are symmetric around $90^\circ$ because collagen fibres are bidirectional. Figure 1 also illustrates the advantage of representing the collagen fibres as a tensorial field: the matrix-vector multiplication ($\hat{\textbf{M}}\textbf{b}$) directly reorients the cell’s migratory direction without the need for additional assumptions to select between the two directions (see Supplementary Information Section S1), due to the bidirectionality of collagen fibres.

#### Cell proliferation

Following previous work (Tarle et al. 2015), we implement a model of cell density-dependent cell proliferation in which the probability that a cell divides in the time step $[t,t+\Delta {t})$ is given by

10 $$\begin{aligned} P_\textrm{p}(\textbf{X}^i(t);\Delta _0,\Delta t,\sigma )= {\left\{ \begin{array}{ll} \displaystyle \frac{\Delta t}{\Delta _0}\left( 1-\frac{\beta \left( \textbf{X}^i(t); \sigma \right) }{6}\right) , & \quad \beta \le 6,\\ 0, & \quad \text {otherwise,} \end{array}\right. } \end{aligned}$$

where $\Delta _0>0$ is the mean cell cycle length in the absence of crowding, $\beta \left( \textbf{X}^i(t), \sigma \right)$ is the number of cells in the region in which the pairwise interaction force is repulsive, and six is assumed to be the maximum packing number (Hales et al. 2017).

#### Collagen fibre dynamics

We assume that cells evolve the collagen fibres through degradation and secretion. The governing equation for collagen fibres is given by Equation (4b), where $\textbf{g}$ is as follows:

11 $$\begin{aligned} \textbf{g}\left( \textbf{X}^1, \textbf{X}^2, \ldots , \textbf{X}^{N(t)}, \mathbf {\Omega };\mathbf {\Theta }\right) = \sum _{i = 1}^{N(t)}\omega \left( \textbf{X}^i, \textbf{x};\sigma \right) \left[ s\left( 1 - \lambda _1 - \lambda _2\right) \hat{\textbf{u}}_\textrm{ave}^i \left( \hat{\textbf{u}}_\textrm{ave}^i\right) ^\textrm{T} - d\,\mathbf {\Omega }\right] ,\nonumber \\ \end{aligned}$$

where $s>0$ and $d>0$ are the collagen secretion and degradation rates per cell, respectively. The term $\left( 1 - \lambda _1 - \lambda _2\right)$ ensures no fibre secretion when the space is fully occupied by collagen fibres. The secretion of collagen fibres by cell *i* is in the direction of cell *i*’s average velocity, $\textbf{u}_\textrm{ave}^i$, over $[t-m,t]$, where^2^

12 $$\begin{aligned} \textbf{u}_\textrm{ave}^i(\textbf{X}^i, t; m)=\frac{1}{m}\int _{t-m}^{t}\frac{\textrm{d}\textbf{X}^i}{\textrm{d}t'}\textrm{d}t'=\frac{1}{m}\left( \textbf{X}^i(t)-\textbf{X}^i(t-m)\right) . \end{aligned}$$

In writing Equation (11), we assume a cell impacts the distribution of collagen fibres within a distance $\sigma /2$ from its centre, recalling $\sigma$ captures the cell diameter. To reflect this, we take

13 $$\begin{aligned} \omega \left( \textbf{X}^i, \textbf{x},\sigma \right) = {\left\{ \begin{array}{ll} 1-\frac{|\textbf{X}^i-\textbf{x}|}{\sigma /2}, & \quad |\textbf{X}^i-\textbf{x}|\le \sigma /2,\\ 0, & \quad |\textbf{X}^i-\textbf{x}|> \sigma /2. \end{array}\right. } \end{aligned}$$

## Computational implementation of the model

In this paper, we use two distinct two-dimensional rectangular domains, *G*, and final times, *T*, to illustrate the results presented in later Section 4:

- **Setup 1.** $G = [0, 540] \times [0, 540] \, \mu \textrm{m}^2$ and $T = 96$ hours, used to showcase the main features of the model in detail.
- **Setup 2.** $G = [0, 360] \times [0, 360] \, \mu \textrm{m}^2$ and $T = 60$ hours (smaller domain size and longer simulation time for computational efficiency), used to investigate the effects of different collagen fibre initial conditions and cell properties on dynamics.

We impose periodic boundary conditions for the cells, and the domain *G* is discretised as $G_\delta$ with a grid size of $\delta = 2 \, \mu \textrm{m}$, and the numerical time step is set to $\Delta {t}=1$ minute. The initial conditions are listed in Supplementary Information Section S5. The collagen fibre field, $\mathbf {\Omega }$, is discretised on $G_\delta$ as $\mathbf {\Omega }_\delta$. At each time step we:

1. calculate cell *i*’s random motility force $\boldsymbol{\xi }^i$ and cell–cell interaction forces $\sum _{j = 1, j \ne i}^{N(t)}\textbf{F}\left( \textbf{X}^i-\textbf{X}^j\right)$ (see Section 2.2.1);
2. extract $\mathbf {\Omega }_\delta$ at cell *i*’s centre using linear interpolation to obtain the matrix $\hat{\textbf{M}}$ for contact guidance (see Equation (8)).

We then update all cell positions according to Equation (5), and update the discretised collagen fibre field $\mathbf {\Omega }_\delta$ according to Equations (11)–(12). Finally, we iterate over the *N*(*t*) cells to let each cell proliferate with probability $P_\textrm{p}$ defined in Equation (10). The daughter cell is placed a distance $\sigma /2$ away at an angle sampled from the uniform distribution $U[0, 2\pi ]$.

Algorithm 1 provides the pseudocode for the numerical simulation of the model. The differential equations are solved using the forward Euler method (Hairer et al. 1987) with time step $\Delta t$, and the integrals are approximated using the midpoint rule (Zorn 2002). Initial conditions for each simulation in Section 4 are stated and visualised in Supplementary Information (Section S5). Python codes to simulate the model are available at https://github.com/YuanYIN99/ECMcell_ContactGuidance_HybridModel.git

**Algorithm 1:**
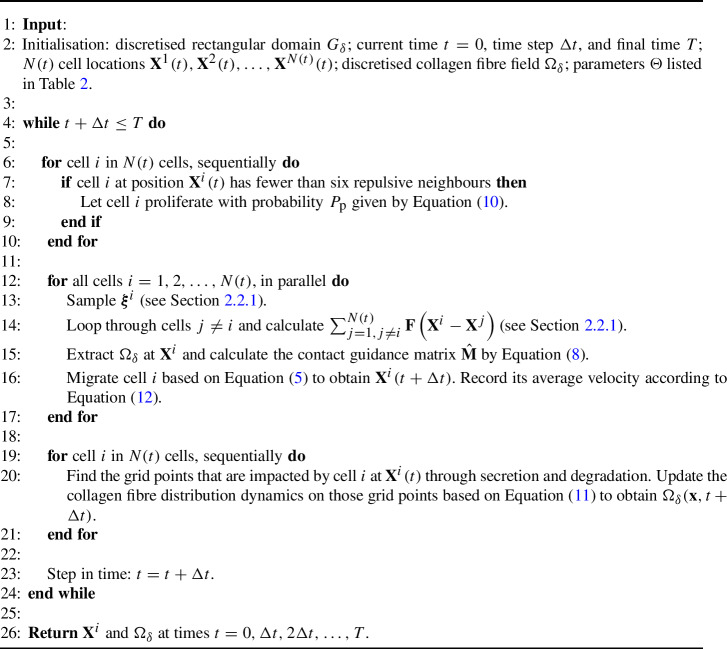
Collective cell migration in a fibrous environment.

## Results

We now demonstrate how differences in cell phenotypes impact patterns of collective cell invasion, underpinned by the interactions between cells and collagen fibres. We first, in Section 4.1, explore the collagen fibre distributions that arise *de novo* from a migratory cell phenotype, before moving to understand how different patterns of collagen fibres drive collective cell invasion in a phenotype that does not re-model the underlying fibre bed in Section 4.2. We then, in Section 4.3, simulate the dynamics of degrading and secreting phenotypes in turn, to tease apart the subtleties of the two-way coupling between cells and collagen fibres. In Section 4.4, we conduct a systematic parameter sweep to reveal how a cell phenotype able to fully re-model collagen via both secretion of new fibres and degradation of old fibres can undergo collective invasion. Finally, in Section 4.5 we showcase the wide range of possible invasion behaviours as the relative contributions of different processes are varied. All cell phenotypes and their corresponding dynamics explored are listed in Table 3.

**Table 3:** Cell phenotypes and their corresponding dynamics explored in Section 4

**Cell phenotypes**	**Section**
Non-collagen modulating phenotype	Section 4.2
Collagen degrading phenotype	Section 4.3
Collagen secreting phenotype	
Collagen-modulating phenotype	Section 4.4
Cell-cell interaction migratory phenotype	Section 4.5
Random motility migratory phenotype	
Contact guidance (in)sensitive phenotype(s)	
Short (& long) memory phenotype(s)	

### Secreted collagen fibre dynamics reflect cell migration patterns

**Fig. 2:**
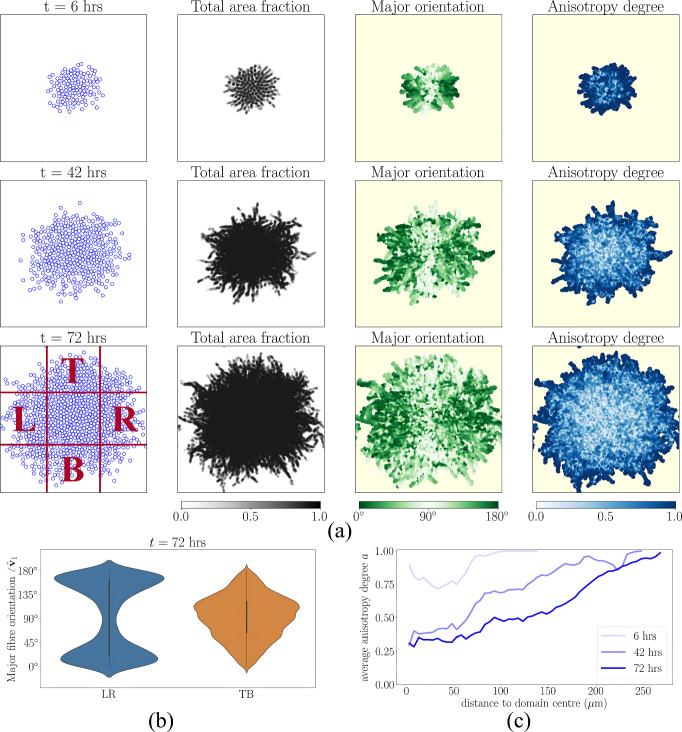
**Secreted collagen fibre dynamics reflect cell migration patterns.** (a) Cell and collagen fibre distributions at 6, 42, and 72 hours. Cells are visualised as blue circles with radius $\sigma /2$. The pale yellow backgrounds denote regions devoid of collagen fibres. (b) Distribution of the angle between $\hat{\textbf{v}}_1$ and the positive *x*-axis in the left and right regions (LR) compared with the top and bottom regions (TB) (the regions ‘L’, ‘R’, ‘T’, and ‘B’ are indicated in the bottom-left of (a)). The violin plot shows the kernel density estimate of this distribution, with a miniature box plot overlaid: the thick black bar represents the interquartile range (IQR, Q1–Q3), the white dot indicates the median, and the thin black lines (‘whiskers’) extend to data points within $1.5\times$IQR from Q1 and Q3. (c) Average anisotropy degree within concentric rings from the domain centre. Simulation details: Setup1 in Section 3. Initially, 100 cells are arranged in a densely packed circular disc at the centre of the domain, forming a confluent arrangement, with no collagen fibres present ($\lambda _1+\lambda _2 = 0$). Collagen fibre secretion and degradation rates are $0.025\text { min}^{-1}$ and $0.0025 \text { min}^{-1}$, respectively

In Figure 2, we showcase how collective cell invasion leads to patterns in collagen fibre dynamics. We take as initial conditions 100 cells clustered in the centre of the domain, with no collagen fibres present. As the cells migrate outwards and proliferate, they lay down collagen fibres that are oriented in the direction of outward expansion. Figure 2(a) shows snapshots of the cell positions, the total area fraction of collagen fibres ($\lambda _1 + \lambda _2$), the major orientation ($\hat{\textbf{v}}_1$), and the anisotropy degree (*a*), shown in grey, green, and blue shading, respectively. Cells at the leading edge exhibit directed motion due to population pressure, resulting in the secretion of highly aligned collagen fibres (Figure 2(b)). Cells behind the front are limited in their motility due to the lack of free space leading to a more isotropic collagen fibre distribution (Figure 2(c)). We note that the dynamics of cells and collagen fibres are qualitatively insensitive to the functional form of the collagen-modulating kernel, $\omega$ (see Figures S4–S5).

### Non-ECM-modulating phenotypes display restricted invasion into dense collagen environments

**Fig. 3:**
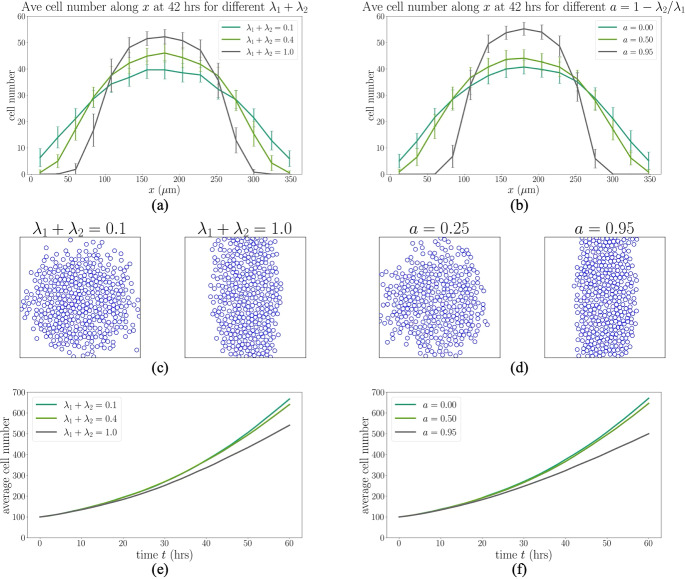
**Non-ECM-modulating phenotypes display restricted invasion into dense collagen environments.** (a),(b) One-dimensional cell density profiles obtained by counting the number of cells along the *x*-direction and averaging over the *y*-direction at 42 hours. Cell density is shown for different total area fractions ($\lambda _1 + \lambda _2$) and anisotropy degrees ($a = 1 - \lambda _2 / \lambda _1$) of collagen fibres. Error bars represent the standard deviation over 40 repetitions. (c),(d) Cell distributions at 42 hours. (e),(f) Average cell number over time. For (a), (c), and (e), $a=0.9$ while for (b), (d), and (f), $\lambda _1+\lambda _2 = 0.8$. In all figures, the major fibre orientation, $\hat{\textbf{v}}_1$, is vertical while the minor orientation, $\hat{\textbf{v}}_2$, is horizontal. Simulation details: Setup2 in Section 3. Initially, 100 cells are arranged in a densely packed circular disc at the centre of the domain, forming a confluent arrangement. Collagen fibre secretion and degradation rates are both set to zero

We now explore collective cell invasion in a non-ECM-modulating phenotype where cells respond to contact guidance cues from the underlying collagen fibres without altering them ($s=d=0$ $\text {min}^{-1}$). Here we again simulate using an initial cluster of 100 cells, but now on a spatially-uniform fibre bed with major fibre orientation, $\hat{\textbf{v}}_1$, vertical and minor fibre orientation, $\hat{\textbf{v}}_2$, horizontal. On the left-hand side of Figure 3, we show results where the collagen fibres are primarily aligned vertically and the total area fraction of collagen varies, whilst on the right-hand side, the anisotropy is varied whilst the total area fraction of collagen is held fixed (again, with primarily vertically aligned collagen fibres).

As expected, Figure 3 shows that both density and anisotropy of the collagen fibre field impact cell invasion patterns. On the left-hand side, where the anisotropy is fixed at $a=0.9$, we see that a low area fraction of collagen fibres does not restrain collective invasion, whereas a high area fraction restricts cell migration horizontally. On the right-hand side of Figure 3, where the total area fraction of collagen fibres is fixed at $\lambda _1+\lambda _2=0.8$, the cell migration patterns reflect the underlying anisotropy of the fibre field (more anisotropy confines cells horizontally).

Another important prediction of the model is that the initial collagen fibre distribution indirectly influences cell proliferation. This occurs because the cell clusters are more compact when the collagen fibres are more dense and more aligned (Figure 3(c),(d), and Figure S2 in the Supplementary Information). This effect is not apparent until approximately 24 hours from the start of the simulation (Figure 3(e),(f)), which corresponds to the average cell cycle length.

### Collagen-modulating phenotypes display different invasion patterns

**Fig. 4:**
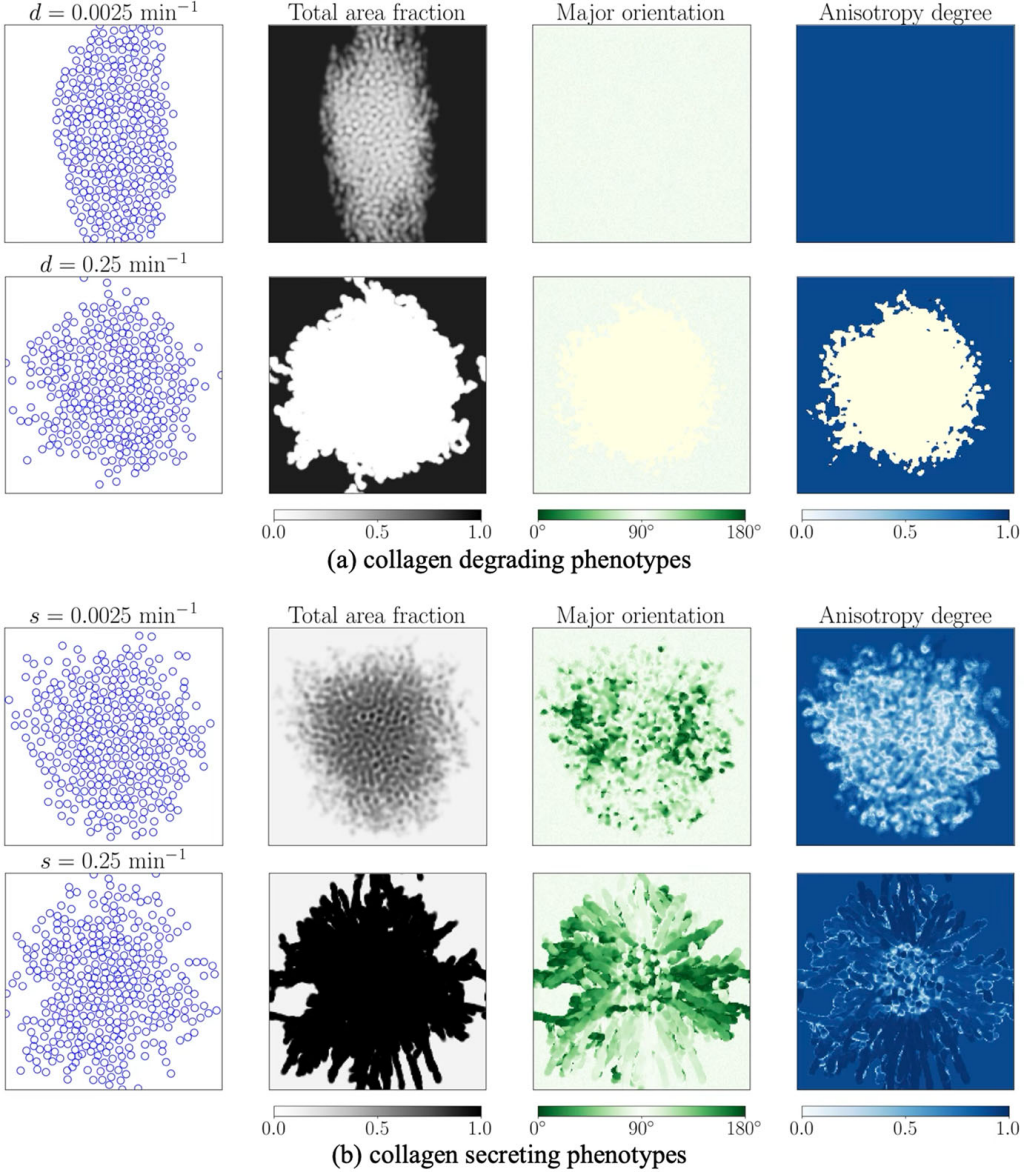
**Dynamics of collagen degrading and secreting phenotypes.** (a) collagen degrading phenotypes display enhanced invasion. Here, the initial conditions have $\lambda _1+\lambda _2=0.9$, $a=1-\lambda _2/\lambda _1=0.9$, vertical $\hat{\textbf{v}}_1$ and horizontal $\hat{\textbf{v}}_2$. (b) collagen-secreting phenotypes promote anisotropic cell invasion and collagen patterns. Here the initial conditions have $\lambda _1+\lambda _2=0.1$, $a=1-\lambda _2/\lambda _1=0.9$, vertical $\hat{\textbf{v}}_1$ and horizontal $\hat{\textbf{v}}_2$. Simulation setup: Setup2 in Section 3. The initial conditions are visualised in Figure S3 in Supplementary Information (Section S5), with 100 cells, and results are displayed at $t=36$ hours. The pale yellow shading denotes a region devoid of collagen fibres

To examine the dynamics of collagen fibre degrading phenotype we set the secretion rate to zero, $s=0$. Figure 4(a) shows that faster collagen fibre degradation reduces contact guidance, with cells displaying invasion patterns similar to when no fibres are present (compare with Figure 2). We observe that a higher degradation rate promotes proliferation by facilitating dispersion of the cells (see Figure S6(a),(b) in Supplementary Information (Section S7)).

We now examine the dynamics of a collagen-secreting phenotype, setting the degradation rate to be zero, $d=0.0$, and the initial total area fraction of collagen to be low so that there is substantial area available into which cells can secrete new collagen. Here, the remodelling process is more intricate, and displays spatial variations across the cluster (see Figure 4(b)). For cells in the top and bottom regions (as defined in Figure 2), migration is primarily aligned with the vertically oriented major fibre direction. In these regions, anisotropy increases over time as newly secreted fibres reinforce this vertical alignment. For cells in the left and right regions (as defined in Figure 2), migration is primarily driven by population pressure arising from cell–cell interactions, and is aligned with the horizontally oriented minor fibre direction. As the cells secrete new collagen, which is primarily horizontally aligned, the fibre anisotropy initially decreases over time as the fibre distribution becomes more uniform (i.e., $\lambda _1 \approx \lambda _2$). It then increases as newly secreted fibres are continually laid down in the horizontal alignment, so that the major fibre direction becomes horizontal. In the central region, the anisotropy decreases over time as cells move predominantly randomly. Therefore, as shown in Figure 4(b), the high collagen-secreting cell phenotype deposits denser collagen fibres in a more anisotropically aligned structure compared with the low-secreting phenotype. Moreover, high secretion rates result in clear major fibre orientations across the domain: horizontal in the left and right regions, and vertical in the top and bottom regions.

### Tensioning secretion and degradation in the phenotype alters invasion patterns

**Table 4:** Secretion and degradation cell phenotypes investigated in Section 4.4

**Label**	**Collagen-related cell phenotypes**
	small secretion with large degradation rates
	large secretion and large degradation rates
	large secretion with small degradation rates

**Fig. 5:**
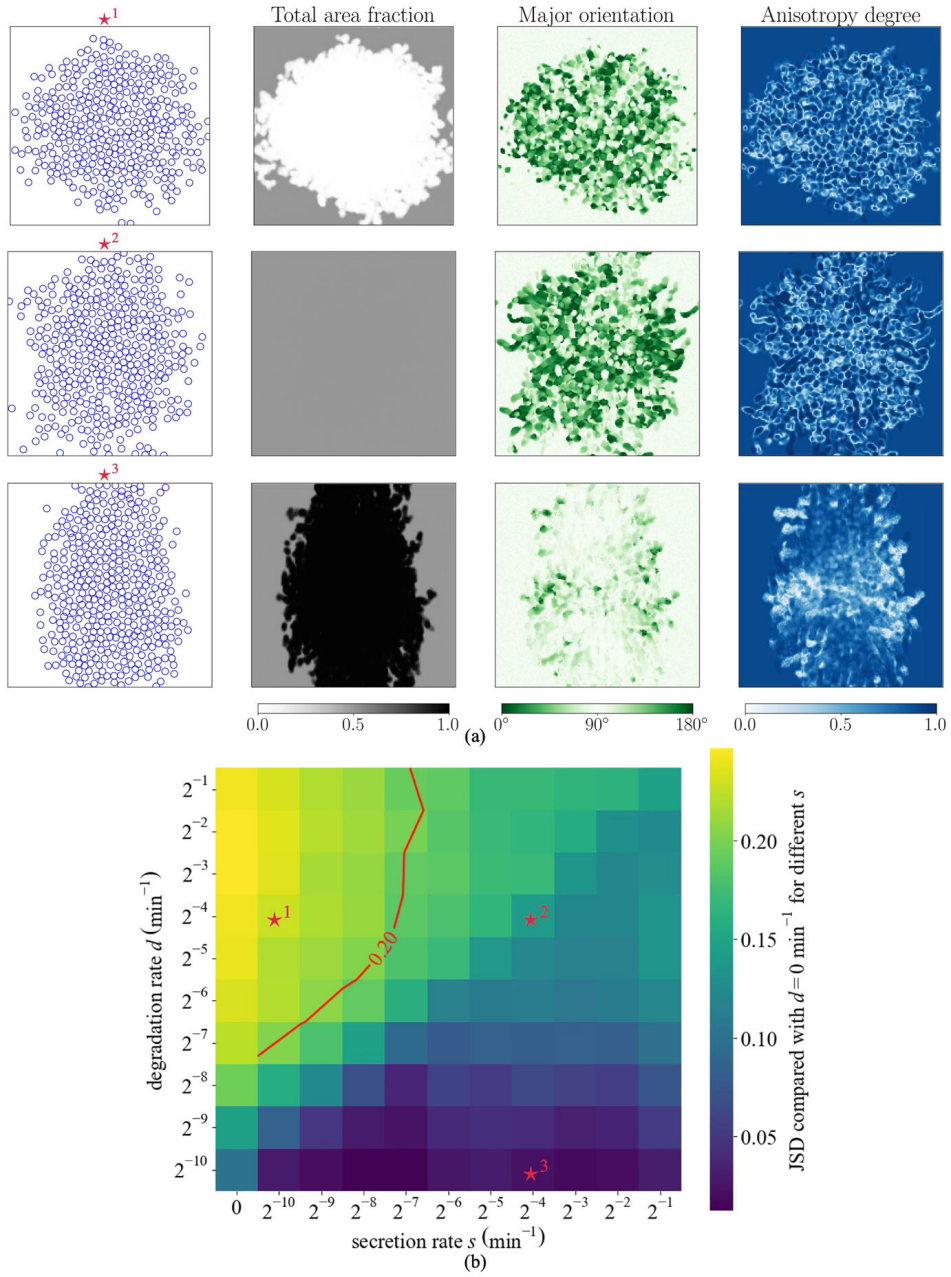
**Tensioning secretion and degradation in the phenotype alters invasion patterns.** Results from a parameter sweep across secretion (*s*) and degradation (*d*) rates. (a) Cell and collagen fibre distributions at 42 hours with : $\left( s, d\right) =\left( 2^{-10}, 2^{-4}\right)$ $\textrm{min}^{-1}$, : $\left( s, d\right) =\left( 2^{-4}, 2^{-4}\right)$ $\textrm{min}^{-1}$, and : $\left( s, d\right) =\left( 2^{-4}, 2^{-10}\right)$ $\textrm{min}^{-1}$, respectively. (b) Heat map showing the similarity of the average cell density along the horizontal direction, compared to that of the non-degrading cell phenotype ($d = 0, \textrm{min}^{-1}$) at 42 hours. The metric used is the Jensen-Shannon Distance (JSD), as explained in the Supplementary Information (Section S4): a value of 0 indicates identical distributions, while 1 indicates complete dissimilarity. Simulation setup: Setup2 in Section 3. The initial conditions are visualised in Figure S3 in Supplementary Information (Section S5), with 100 cells, $\lambda _1+\lambda _2=0.5$, $a=1-\lambda _2/\lambda _1=0.9$, vertical $\hat{\textbf{v}}_1$ and horizontal $\hat{\textbf{v}}_2$

To investigate how a phenotype that both secretes and degrades fibres affects collagen fibre dynamics and cell invasiveness, we perform a parameter sweep across different fibre secretion and degradation rates. We consider a scenario in which there is very strong contact guidance initially due to a high area fraction of aligned collagen. Figure 5(a) illustrates the cell and collagen fibre distributions for small secretion with large degradation rates (), large secretion and large degradation rates (), and large secretion with small degradation rates (), see Table 4. For a cell phenotype that predominantly degrades collagen (), the initial collagen distribution is degraded and becomes isotropically distributed, reducing the strength of contact guidance while facilitating cell invasion in all directions. This is consistent with results in Figure 4(a). For a cell phenotype that equally secretes and degrades collagen (), the initially aligned fibres are degraded: this results in reduced contact guidance so that newly secreted collagen is relatively isotropic. Comparing the  phenotype with , we observe that migration is facilitated in all directions, with a stronger tendency in the vertical direction. This arises because the higher secretion rate of the  phenotype produces denser collagen fibres than the  phenotype. However, the large degradation rate of the  phenotype subsequently leads to a weakening of these vertical cues, thereby eventually facilitating horizontal migration. Finally, for a cell phenotype that predominantly secretes collagen (), the initial anisotropy, and thus the contact guidance cues, are reinforced in the top and bottom regions. This occurs because cells in these regions tend to migrate vertically due to population pressure, even in the absence of contact guidance. This promotes enhanced invasion along the vertical axis, while horizontal invasion is suppressed because guidance cues from population pressure are in tension with those from the collagen distribution. This is consistent with results in Figure 4(b).

We also examine, given strong vertical contact guidance cues initially, how dominant the degradation phenotype must be for cells to effectively modulate the collagen fibre distribution and achieve isotropic invasion. To this end, we compare one-dimensional cell density profiles, generated by averaging the cell density in the *y*-direction, given different collagen degradation rates. The metric we use to compare these density profiles is the Jensen-Shannon distance (JSD), which is a bounded measure of how far apart two distributions are, with smaller values of the JSD indicating higher similarity (see Supplementary Information Section S4). Here, we take $\textrm{JSD} = 0.2$ as the threshold beyond which invasion is substantially different from the corresponding pattern observed with the same level of secretion but no degradation. Figure 5(b) shows that once the secretion rate exceeds a critical value ($s = 2^{-7}$ $\textrm{min}^{-1}$), the cell population cannot overcome the initial vertical contact guidance cues no matter how rapid the rate of degradation.

### Showcase of invasion patterns across a range of different phenotypes

**Fig. 6:**
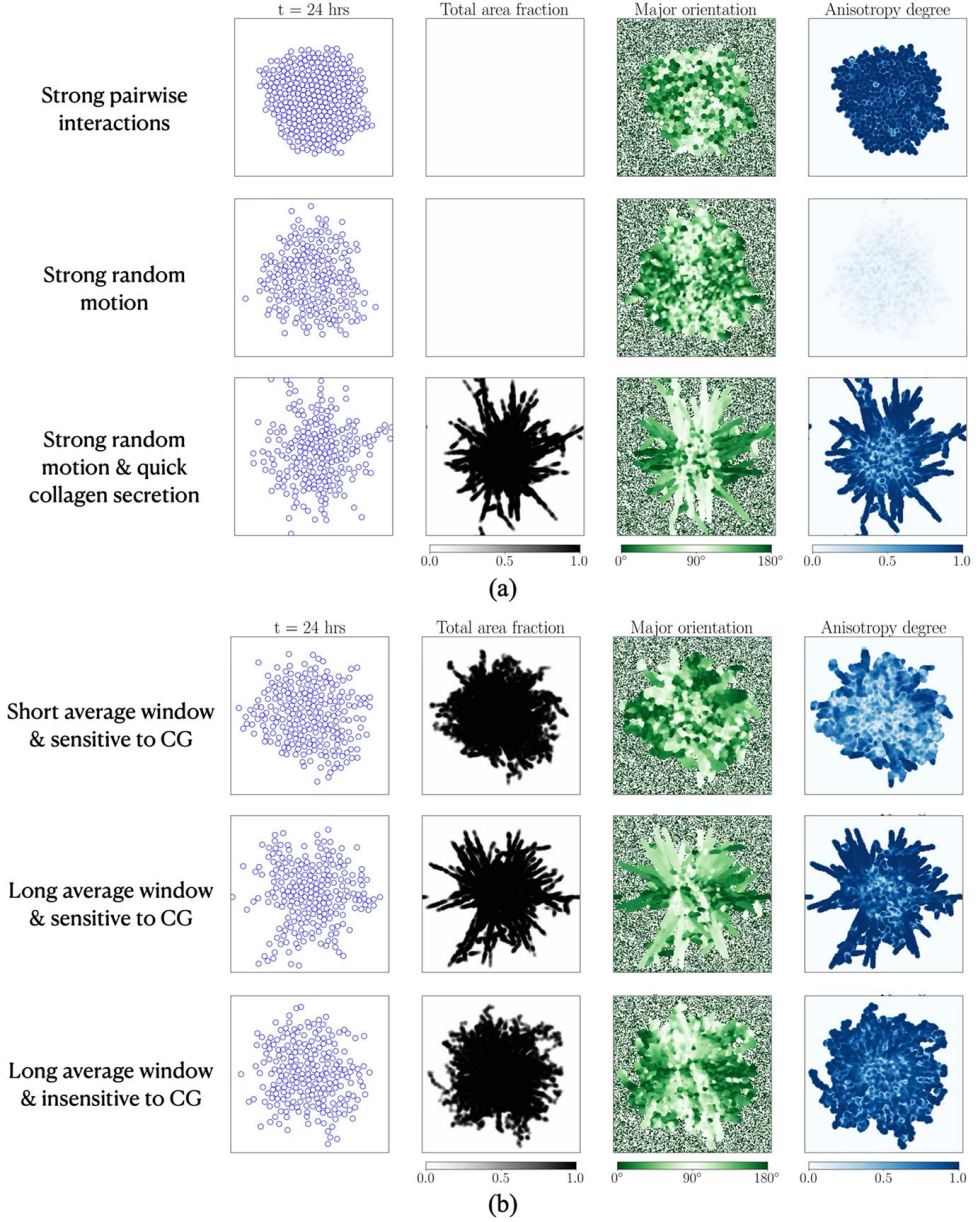
**Showcasing the range of invasion patterns arising from different cell phenotypes.** Cell and collagen fibre distributions at 24 hours are shown for: (a) varying motility parameters, with $(D, \epsilon ) = (0.075, 0.05)$ $\text {}\mu \text {m}^2\text { min}^{-1}$ (top row), and $(D, \epsilon ) = (0.3, 0.05)$ $\text {}\mu \text {m}^2\text { min}^{-1}$ (middle and bottom rows); (b) varying contact guidance (CG) responses, with $(m, \bar{\lambda }) = (1\text { min}, 0.005)$ (top row), $(m, \bar{\lambda }) = (1200\text { min}, 0.005)$ (middle row), and $(m, \bar{\lambda }) = (1200\text { min}, 0.9)$ (bottom row). The collagen degradation rate is fixed at $d=0.0005\text { min}^{-1}$ for all cases, while the secretion rate is $s=0.05\text { min}^{-1}$, except in the first two rows of (a), where $s=5.05\times 10^{-6}\text { min}^{-1}$ to ensure that the total fibre density remains constant during invasion. Simulation setup: Setup2 in Section 3. Initial conditions are visualised in Figure S3 in the Supplementary Information (Section S5), with 100 cells, $\lambda _1 + \lambda _2 = 0.01$, and $a = 1 - \lambda _2 / \lambda _1 = 0$, corresponding to uniformly randomly distributed collagen fibres

Finally, by varying specific parameter values listed in Table 2, we showcase the wide range of cell invasion and collagen fibre patterns that can arise due to different cell phenotypes. Figure 6(a) demonstrates that when cell motility is primarily driven by pairwise interactions rather than random motion, the colony remains compact with a high degree of radial symmetry. Because the cell motility is less stochastic in nature, the secreted collagen fibres exhibit a high degree of alignment. In contrast, when cell migration is dominated by random motility, the resulting cell cluster is more dispersed, and the secreted fibres appear less aligned. Another key observation is that finger-like invasion patterns emerge only when cells secrete collagen fibres sufficiently quickly (bottom row of Figure 6(a)). In this regime, cells at the edge of the cluster show a high degree of persistence, reinforcing both the orientation and anisotropy of the secreted fibres. This, in turn, further guides the direction of cell migration.

Figure 6(b) shows that varying the length of the averaging window (which can be referred to as the memory window, and determines the alignment of secreted collagen fibres) significantly influences the patterns of cell invasion. A longer averaging window (i.e. longer memory) smooths out the effects of random motion and results in the secretion of more aligned and spatially organised collagen fibres, which in turn enhances the emergence of a finger-like invasion pattern. On the other hand, a short averaging window (i.e. short memory) leads to more random motility, and less well aligned collagen fibres. Additionally, the sensitivity of cells to contact guidance cues from the underlying collagen fibres plays a crucial role in shaping the patterns of cell invasion. If cells only respond to contact guidance cues when the collagen fibre area fraction is high (i.e. large $\bar{\lambda }$) then, despite an aligned and structured fibre field (due to the long averaging window), the cells do not exhibit finger-like behaviour.

## Discussion

In this study, we developed a computationally tractable mathematical model to investigate the role of ECM-generated contact guidance in directing collective cell migration. The model incorporates biologically realistic cell–cell interactions, including volume filling and cell–cell adhesion, while focusing on the reciprocal influence between migrating cells and the orientation of collagen fibres. Through an extensive parameter sweep, we systematically examined how variations in cell phenotype impact collective invasion behaviours within the ECM. A key advantage of the representation of the ECM as a tensorial field is the ease with which we can account for its density- and anisotropy-dependent contact guidance effects on cells.

Our model demonstrates several key findings. Firstly, when considering a region initially devoid of collagen fibres, collective cell invasion generates distinct patterns in collagen fibre deposition, with migrating cells secreting fibres in the direction of outward expansion. Cells at the leading edge of the population produce highly aligned fibres due to directed movement, while restricted motility behind the front due to a lack of free space results in a more isotropic fibre distribution. Secondly, in non-ECM-modulating phenotypes, where cells respond to but do not remodel collagen fibres, both collagen fibre area fraction and anisotropy significantly affect collective invasion patterns. A higher collagen area fraction restricts cell migration in the minor fibre direction, while increased fibre anisotropy further confines invasion along the dominant fibre orientation. These matrix properties indirectly influence cell proliferation, as denser and more aligned collagen environments lead to more compact cell clusters. Thirdly, collagen-degrading phenotypes reduce contact guidance by degrading fibres, resulting in more dispersed invasion patterns and enhanced proliferation. In contrast, collagen-secreting phenotypes create spatially varied invasion behaviours, with the direction of cell migration in the peripheral regions either more aligned with the major or minor fibre direction of the initial collagen bed, depending on their location. As a result, the cells generate complex, region-specific changes in fibre anisotropy as new collagen is deposited. Varying the balance of collagen secretion and degradation rates significantly alters invasion patterns and collagen fibre dynamics. When degradation dominates, fibres are rapidly removed, enabling uniform invasion, while high secretion rates lead to a reinforcement of existing anisotropy, directing invasion along preferred orientations. Finally, a wide range of different invasion patterns can be established through variation of the relative strengths of cell–cell interactions and random motility, as well as the extent to which cells respond to contact guidance cues.

Our model investigates the role of contact guidance in collective cell invasion using a general and modular approach, allowing for easy extensions and applications to specific biological processes. For instance, to explore wound healing, we could additionally model the dynamics of relevant chemical signals and the response of cells (e.g. chemotaxis) to them. It is also possible to extend the model to include populations of cells of mixed phenotype, and switching of cells between different phenotypes, as observed in a range of systems (e.g., Rognoni et al. (2018); Crossley et al. (2024)). Recent experiments also suggest that collagen fibres can mature over time, becoming increasingly resistant to remodelling during tumour progression (Fang et al. 2014). Modelling such behaviours would involve tracking the time history of the collagen fibres. Furthermore, collagen types I and III are known to have distinct roles in tissue injury and regeneration (Singh et al. 2023), and we could exploit the model to track different collagen subtypes.

## Supplementary Information

Below is the link to the electronic supplementary material.

Supplementary file 1 (PDF 5747 KB)

## Footnotes

1 This means that the two orientations of a fibre, separated by $180^{\circ }$, are indistinguishable.

2 We set $\textbf{X}^i(t-m)=\textbf{X}^i(0)$ if $t<m$, where $\textbf{X}^i(0)$ is the initial position for cell $i = 1,...,N.$.
