# Supplementary material for: The influence of cell phenotype on collective cell invasion into the extracellular matrix
Source: Bull Math Biol. 2025 Dec 26;88(1):13. doi: 10.1007/s11538-025-01560-9 (PMC12743096; doi:10.1007/s11538-025-01560-9)
Supplement: Supplementary file 1 — (PDF 5747 KB) [file 11538_2025_1560_MOESM1_ESM.pdf]

Supplementary Information

S1 Interpretation of $\boldsymbol{\Omega}$ and matrix normalisation

Suppose the major fibre orientation $\hat{\mathbf{v}}_1$ makes an angle θ_1 with the positive x-axis, $\hat{\mathbf{v}}_1 = (\cos(\theta_1), \sin(\theta_1))^{\mathrm{T}}$. As $\hat{\mathbf{v}}_2$ is orthonormal to $\hat{\mathbf{v}}_1$, $\hat{\mathbf{v}}_2 = (-\sin(\theta_1), \cos(\theta_1))^{\mathrm{T}}$. Based on Equation (2) of the main text we have

$$
\begin{aligned}
\boldsymbol{\Omega} &= \lambda_1 \hat{\mathbf{v}}_1 \hat{\mathbf{v}}_1^{\mathrm{T}} + \lambda_2 \hat{\mathbf{v}}_2 \hat{\mathbf{v}}_2^{\mathrm{T}} \\
&= \begin{bmatrix} | & | \\ \hat{\mathbf{v}}_1 & \hat{\mathbf{v}}_2 \\ | & | \end{bmatrix} \begin{bmatrix} \lambda_1 & 0 \\ 0 & \lambda_2 \end{bmatrix} \begin{bmatrix} — & \hat{\mathbf{v}}_1 & — \\ — & \hat{\mathbf{v}}_2 & — \end{bmatrix} \\
&= \underbrace{\begin{bmatrix} \cos(\theta_1) & -\sin(\theta_1) \\ \sin(\theta_1) & \cos(\theta_1) \end{bmatrix}}_{\substack{:=\mathbf{P} \\ \text{anticlockwise rotation of } \theta_1}} \underbrace{\begin{bmatrix} \lambda_1 & 0 \\ 0 & \lambda_2 \end{bmatrix}}_{\substack{:=\mathbf{D} \\ \text{rescale and re-orient}}} \underbrace{\begin{bmatrix} \cos(\theta_1) & \sin(\theta_1) \\ -\sin(\theta_1) & \cos(\theta_1) \end{bmatrix}}_{\substack{:=\mathbf{P}^{\mathrm{T}} \\ \text{clockwise rotation of } \theta_1}} .
\end{aligned} \tag{14}
$$

For any vector $\mathbf{b}$, $\boldsymbol{\Omega}\mathbf{b}$: (i) rotates $\mathbf{b}$ clockwise by θ_1 and obtains $\mathbf{P}^{\mathrm{T}}\mathbf{b}$; (ii) rescales the first and the second elements of $\mathbf{P}^{\mathrm{T}}\mathbf{b}$ by λ_1 and λ_1, respectively, and obtains $\mathbf{D}\mathbf{P}^{\mathrm{T}}\mathbf{b}$. Unless the collagen fibres are isotropic $\lambda_1 = \lambda_2$, the rescaling also leads to re-orientation of an angle θ'; (iii) rotates $\mathbf{D}\mathbf{P}^{\mathrm{T}}\mathbf{b}$ anticlockwise by θ_1 and obtains $\mathbf{P}\mathbf{D}\mathbf{P}^{\mathrm{T}}\mathbf{b} = \boldsymbol{\Omega}\mathbf{b}$. Step (ii) breaks the length-preserving property, therefore in order to obtain $\hat{\boldsymbol{\Omega}}$ such that $|\hat{\boldsymbol{\Omega}}\mathbf{b}| = |\mathbf{b}|$, we define

$$
\hat{\boldsymbol{\Omega}} = C_{\mathbf{b}}(\boldsymbol{\Omega})\boldsymbol{\Omega}, \quad \text{where } C_{\mathbf{b}}(\boldsymbol{\Omega}) = |\mathbf{b}|/|\boldsymbol{\Omega}\mathbf{b}|. \tag{15}
$$

Equation (15) defines the normalisation of $\boldsymbol{\Omega}$ and Figure S1 visualises how $\hat{\boldsymbol{\Omega}}$ reorients a vector $\mathbf{b}$. Similarly, to normalise $\mathbf{M}$, as defined in Equation (8) of the main text, we have $\hat{\mathbf{M}} = |\mathbf{b}|/|\mathbf{M}\mathbf{b}|$ for any vector $\mathbf{b}$.

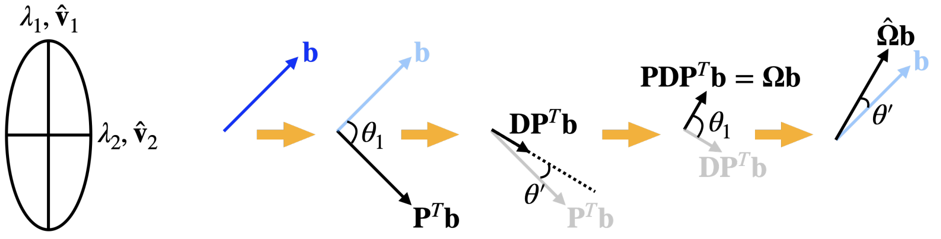

Figure S1: **Schematic diagram of $\hat{\boldsymbol{\Omega}}\mathbf{b}$** given $\boldsymbol{\Omega} = \lambda_1 \hat{\mathbf{v}}_1 \hat{\mathbf{v}}_1^{\mathrm{T}} + \lambda_2 \hat{\mathbf{v}}_2 \hat{\mathbf{v}}_2^{\mathrm{T}}$ and $\hat{\boldsymbol{\Omega}}$ as defined in Equation (15).

S2 The ranges for $\lambda_{1,2}$ and $\lambda_1 + \lambda_2$

The total area fraction occupied by collagen fibres ($\lambda_1 + \lambda_2$) lies between zero and one, as

$$
\begin{aligned}
\lambda_1 + \lambda_2 &= \mathrm{tr}(\mathbf{\Omega}) \qquad \text{[as } \lambda_{1,2} \text{ are eigenvalues]} \\
&= \mathrm{tr}\left(\frac{1}{\pi}\int_0^\pi \hat{\mathbf{u}}(\theta)\hat{\mathbf{u}}^{\mathrm{T}}(\theta)\rho(\theta,\mathbf{x},t)\mathrm{d}\theta\right) \qquad \text{[by Equation (1)]} \\
&= \frac{1}{\pi}\int_0^\pi tr\left(\hat{\mathbf{u}}(\theta)\hat{\mathbf{u}}^{\mathrm{T}}(\theta)\right)\rho(\theta,\mathbf{x},t)\mathrm{d}\theta \\
&= \frac{1}{\pi}\int_0^\pi \rho(\theta,\mathbf{x},t) \qquad \text{[as } \hat{\mathbf{u}} \text{ is a unit vector]} \\
&\in [0,1] \qquad \text{[by the definition of } \rho \in [0,1]\text{]}.
\end{aligned}
\tag{16}
$$

Moreover, as $\mathbf{\Omega}$ is a symmetric positive semi-definite matrix (see Equation (2)), $\lambda_{1,2} \in [0,1]$.

S3 Average number of repulsive neighbours per cell

Figure S2 shows the average number of cells within the repulsive range of $2^{1/6}\sigma$ of a given cell, where σ represents the cell diameter. We observe that denser and more anisotropic collagen fibres result in more compact cell clusters, causing each cell to have more repulsive neighbours, thus less space to proliferate.

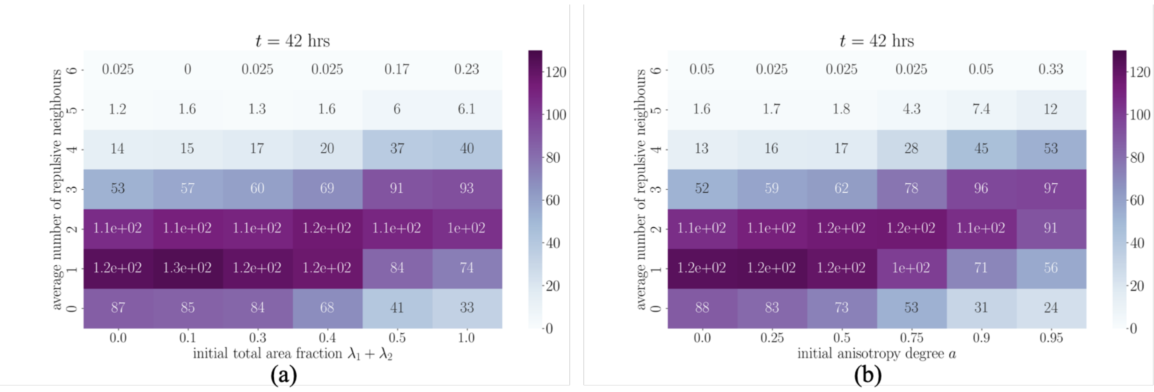

Figure S2: **Average number of repulsive neighbours per cell** given no secretion and degradation. Results are averaged over 40 simulations, given different total area fractions ($\lambda_1 + \lambda_2$) and anisotropy degrees ($a = 1 - \lambda_2/\lambda_1$) of collagen fibres.

S4 Jensen-Shannon distance (JSD)

To quantify the similarity between two distributions, we use the Jensen-Shannon distance (JSD). The JSD between two probability vectors $\mathbf{p}$ and $\mathbf{q}$ is defined as

$$\mathrm{JSD}(\mathbf{p}, \mathbf{q}) := \sqrt{\frac{D(\mathbf{p} \parallel \mathbf{m}) + D(\mathbf{q} \parallel \mathbf{m})}{2}}, \tag{17}$$

where $\mathbf{m}$ is the point-wise mean of $\mathbf{p}$ and $\mathbf{q}$, and $D(A \parallel B) = \sum_{x \in \mathcal{X}} A(x) \log \left(A(x)/B(x) \right)$ is the Kullback-Leibler divergence. The JSD is symmetric and bounded between $[0, 1]$, and the similarity between two distributions is greater for JSD closer to zero, and vice versa.

S5 Initial conditions for simulations

In all simulations, 100 cells are initially arranged in a densely packed circular disc at the centre of the domain with radius $60\,\mu$m, forming a confluent configuration. For Figure 2 in the main text, we initialise the system without any collagen fibres. For Figure 3(a), (c), and (e), the collagen fibres are initialised with a high anisotropy degree ($a = 0.9$), while the total fibre density ($\lambda_1 + \lambda_2$) is varied among 0.1, 0.4, and 1. For Figure 3(b), (d), and (f), the total fibre density is fixed at $\lambda_1 + \lambda_2 = 0.8$, while the anisotropy degree is varied among 0.0, 0.5, and 0.95. In Figure 4(a), collagen fibres are initialised with a high density ($\lambda_1 + \lambda_2 = 0.9$), whereas in Figure 4(b), a low density ($\lambda_1 + \lambda_2 = 0.1$) is used. In both cases, the fibres are aligned ($a = 0.9$). For Figure 5, the collagen fibres are initialised with $\lambda_1 + \lambda_2 = 0.5$ and $a = 0.9$. In Figure 6, a sparse and isotropic collagen distribution is used, with $\lambda_1 + \lambda_2 = 0.01$ and $a = 0$. For Figures 2–5, the angle between the major fibre orientation and the positive x-axis, denoted $\angle\hat{\mathbf{v}}_1$, is sampled from the interval $\pi/2 + U[-\pi/18, \pi/18]$. In contrast, for Figure 6, the collagen fibres are randomly oriented, with $\angle\hat{\mathbf{v}}_1$ sampled uniformly from $U[-\pi, \pi]$. All initial conditions, except for those in Figure 3, are visualised in Figure S3.

S6 Different collagen modulating kernels ω lead to similar dynamics

Recall that the collagen-modulating kernel ω, defined in Equation (2), characterises the strength and range of collagen degradation and secretion. We now investigate whether the cell and collagen fibre dynamics are sensitive to the functional form of ω. Figure S4 illustrates cell

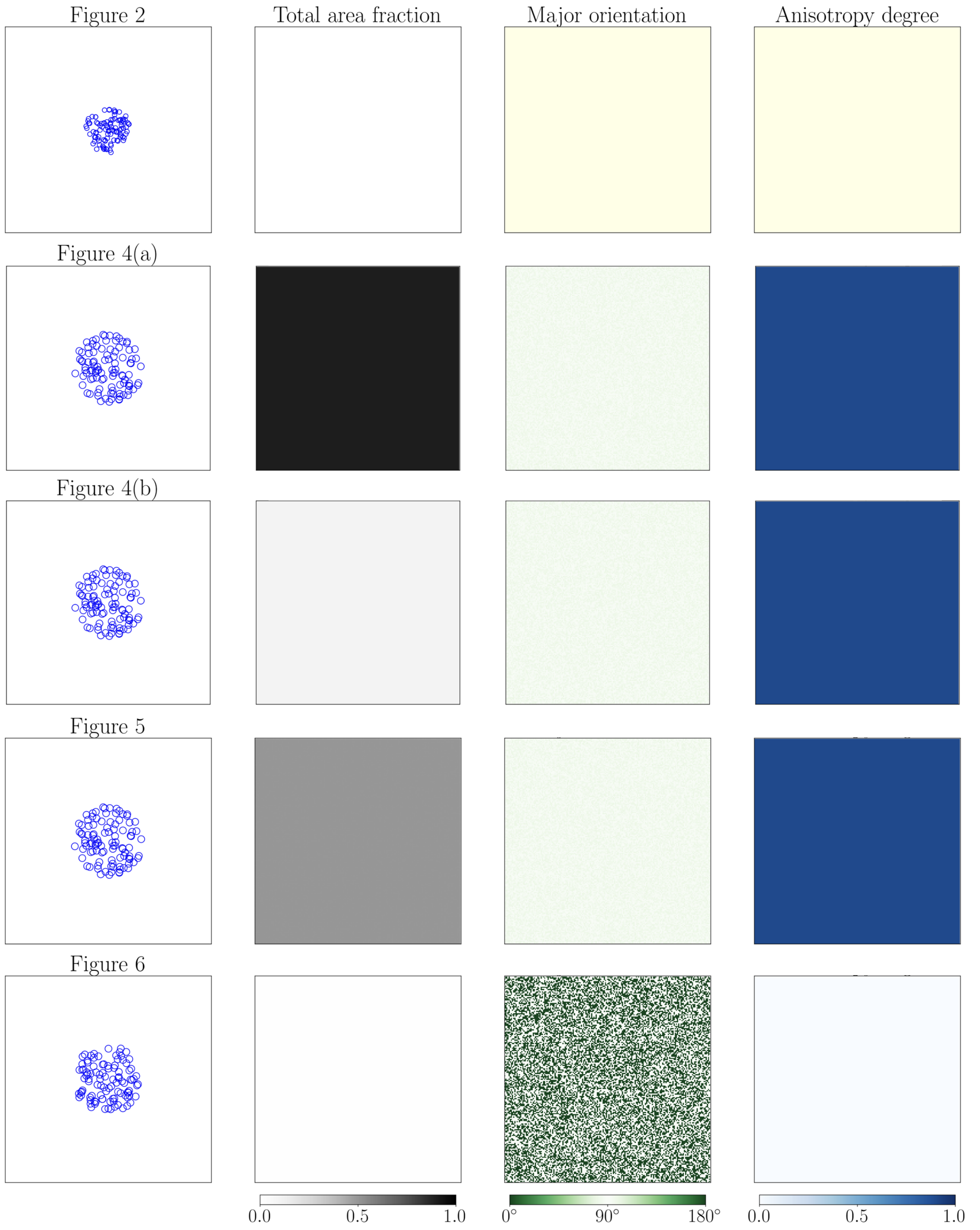

Figure S3: **Initial conditions for results in the main text.** 100 cells are initially arranged in a densely packed circular disc at the centre of the domain with radius 60 μm, forming a confluent configuration. Pale yellow backgrounds indicate regions devoid of collagen fibres. Simulation details: Setup1 with a larger domain and longer simulation time is used for Figure 2, while Setup2 is used for Figures 3–6.

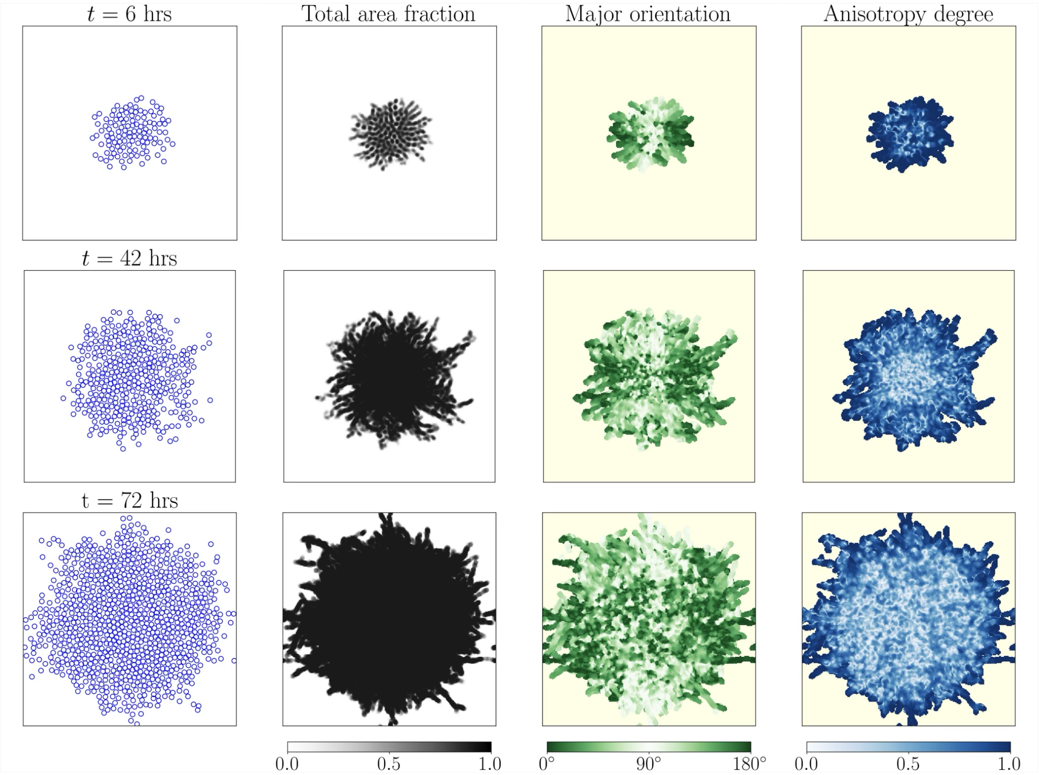

Figure S4: **Cell migration patterns are shown when the collagen-modulating kernel ω is taken as a Heaviside function with support $\sigma/2$, corresponding to the cell radius.** This figure can be compared with Figure 2 in the main text.

migration and collagen fibre dynamics when ω is taken to be a Heaviside function:

$$\omega\left(\mathbf{X}^i, \mathbf{x}, \sigma\right) = \begin{cases} 1, & |\mathbf{X}^i - \mathbf{x}| \leq \sigma/2, \\ 0, & |\mathbf{X}^i - \mathbf{x}| > \sigma/2. \end{cases} \tag{18}$$

We note that since $\sigma/2$ is a measure of the cell radius, this functional form is intended to restrict secretion and degradation locally to a cell. On the other hand, Figure S5 shows the cell migration and collagen fibre dynamics for

$$\omega\left(\mathbf{X}^i, \mathbf{x}, \sigma\right) = \begin{cases} 1 - \frac{|\mathbf{X}^i - \mathbf{x}|}{\sigma}, & |\mathbf{X}^i - \mathbf{x}| \leq \sigma, \\ 0, & |\mathbf{X}^i - \mathbf{x}| > \sigma. \end{cases} \tag{19}$$

In this case the range over which collagen is secreted and degraded is doubled to σ. Comparing Figures S4–S5 with Figure 2, we observe no qualitative differences in cell or collagen fibre dynamics.

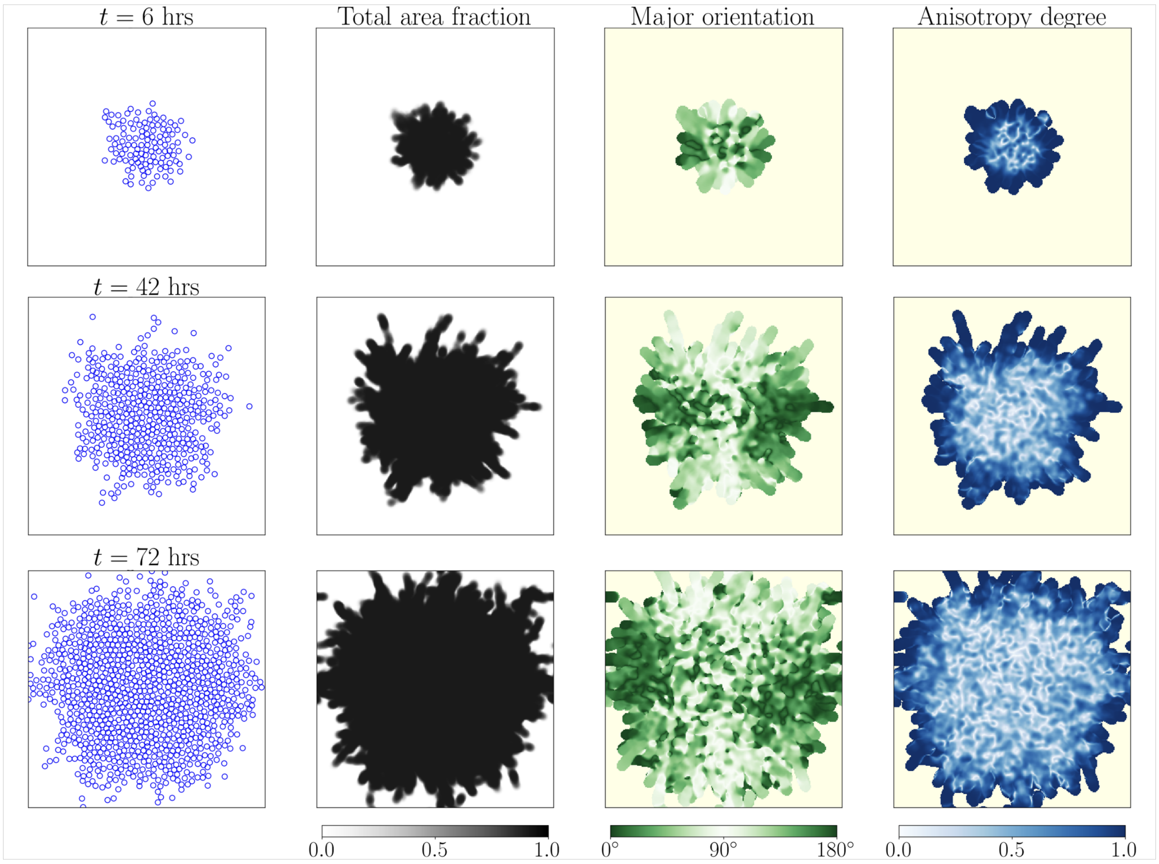

Figure S5: **Cell migration patterns are shown when the range of collagen secretion and degradation extends beyond the space occupied by a single cell; that is, the range of collagen modulation is σ, with $\sigma/2$ representing the cell radius.** This figure can be compared with Figure 2 in the main text.

S7 Collagen degrading phenotypes display different invasion patterns

Figure S6(a) compares the one-dimensional cell density profiles averaged along the y-direction, for slow ($d = 0.0025$ min^{-1}) and fast ($d = 0.25$ min^{-1}) collagen degradation. The results show that dense, vertically aligned collagen fibres restrict horizontal cell invasion, leading to a more compact, vertically striped cell pattern. Consequently, cell density-dependent proliferation is more strongly suppressed in the case of fast degradation ($d = 0.25$ min^{-1}), as shown in Figure S6(b).

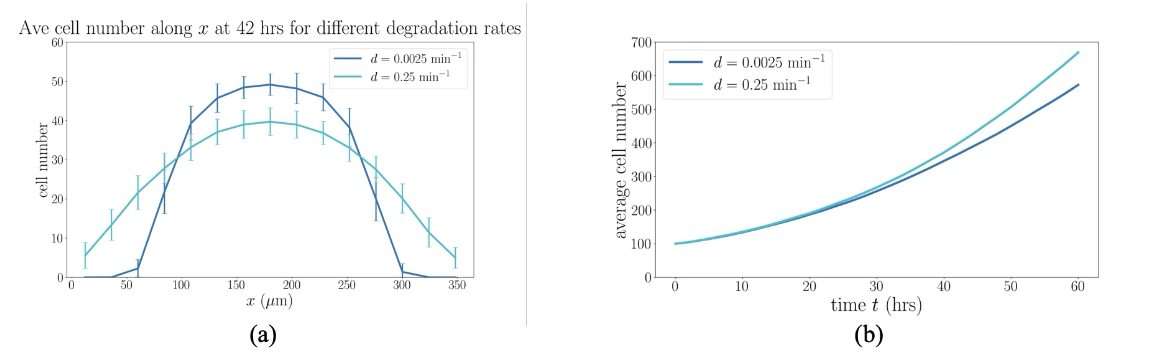

(a) (b)

Figure S6: **Collagen degrading phenotypes enhance horizontal invasion.** (a) One-dimensional cell density profiles obtained by counting the number of cells along the x-direction and averaging over the y-direction for $d = 0.0025$ min^{-1} and $d = 0.25$ min^{-1} at 42 hours. Error bars represent the standard deviation over 40 repetitions. (b) Average cell number over time for $d = 0.0025$ min^{-1} and $d = 0.25$ min^{-1}. This figure is related to Figure 4(a) in the main text.
